# Engineered Full Thickness Electrospun Scaffold for Esophageal Tissue Regeneration: From In Vitro to In Vivo Approach

**DOI:** 10.3390/pharmaceutics14020252

**Published:** 2022-01-21

**Authors:** Silvia Pisani, Stefania Croce, Simone Mauramati, Marta Marmonti, Lorenzo Cobianchi, Irene Herman, Rossella Dorati, Maria Antonietta Avanzini, Ida Genta, Marco Benazzo, Bice Conti

**Affiliations:** 1 Otorhinolaryngology Unit, Department of Surgical Sciences, Fondazione IRCCS Policlinico San Matteo, 27100 Pavia, Italy; s.mauramati@smatteo.pv.it (S.M.); i.herman@smatteo.pv.it (I.H.); m.benazzo@smatteo.pv.it (M.B.); 2 Department of Clinical, Surgical, Diagnostic & Pediatric Sciences, Fondazione IRCCS Policlinico San Matteo, 27100 Pavia, Italy; s.croce@smatteo.pv.it (S.C.); l.cobianchi@smatteo.pv.it (L.C.); 3 Department of Drug Sciences, University of Pavia, 27100 Pavia, Italy; marta.marmonti01@universitadipavia.it (M.M.); rossella.dorati@unipv.it (R.D.); ida.genta@unipv.it (I.G.); bice.conti@unipv.it (B.C.); 4 Department of Paediatric Oncoaematology, IRCCS Policlinico S. Matteo, 27100 Pavia, Italy; ma.avanzini@smatteo.pv.it

**Keywords:** tissue engineering, electrospun nanofibers, esophagus regeneration, tubular scaffold, mesenchymal stem cell, in vivo implantation

## Abstract

Acquired congenital esophageal malformations, such as malignant esophageal cancer, require esophagectomy resulting in full thickness resection, which cannot be left untreated. The proposed approach is a polymeric full-thickness scaffold engineered with mesenchymal stem cells (MSCs) to promote and speed up the regeneration process, ensuring adequate support and esophageal tissue reconstruction and avoiding the use of autologous conduits. Copolymers poly-L-lactide-co-poly-ε-caprolactone (PLA-PCL) 70:30 and 85:15 ratio were chosen to prepare electrospun tubular scaffolds. Electrospinning apparatus equipped with two different types of tubular mandrels: cylindrical (∅ 10 mm) and asymmetrical (∅ 10 mm and ∅ 8 mm) were used. Tubular scaffolds underwent morphological, mechanical (uniaxial tensile stress) and biological (MTT and Dapi staining) characterization. Asymmetric tubular geometry resulted in the best properties and was selected for in vivo surgical implantation. Anesthetized pigs underwent full thickness circumferential resection of the mid-lower thoracic esophagus, followed by implantation of the asymmetric scaffold. Preliminary in vivo results demonstrated that detached stitch suture achieved better results in terms of animal welfare and scaffold integration; thus, it is to be preferred to continuous suture.

## 1. Introduction

Congenital esophageal malformations, such as atresia, and acquired ones, such as chronic gastroesophageal reflux, Barrett’s esophagus, and strictures, often require surgical intervention and esophageal reconstruction [1]. In particular, malignant esophageal cancer requires endoscopic mucosal resection (EMR) or esophagectomy, resulting in a partial thickness or full-thickness resection, respectively. EMR has become an accepted treatment for early-stage esophageal cancer and high-grade dysplasia associated with Barrett’s esophagus. Instead, higher grade and more invasive lesions typically require complete esophagus resection (esophagectomy) that cannot be left untreated because it is mandatory to maintain gastrointestinal continuity. Traditionally, the resected tract is replaced with an autologous conduit (such as gastric, jejunal, and colonic interpositions), but this procedure is associated with significant morbidity, rupture of suture or stenosis, and a mortality rate of about 4% [2]. Moreover, esophagectomy is a very invasive surgery that could induce an intense systemic inflammatory response (SIR), stimulating the release of proinflammatory cytokines, which may increase postsurgical risk of cancer recurrence [3]. Another serious possible surgical complication after esophagectomy is postoperative pneumonia (whose frequency is reported to be 7.6–35.9%), which has been a major cause of hospital mortality (for about 2.7–8.7%) [4]. Problems related to the function of the GI tract such as acid reflux, delayed esophageal transplantation, or dumping syndrome can also invalidate the surgical outcome [5]. Therefore, studies on regenerative approaches aimed at improving esophageal reconstructive techniques are ongoing. Engineering artificial esophageal scaffolds similar to native esophagus are emerging alternatives that are able to permit bolus and liquids transit, possess mechanical characteristics suitable to withstand leak or rupture (stress and strains that must be endured are approximately 0.2–1 MPa and 175% elongation), and guarantee expansion from the resting collapse state to a dilated state [6,7]. Biomaterial selection is a critical aspect for a successful scaffold able to sustain esophageal reconstruction. Synthetic polymers such as polyurethane (PU), polylactic acids (PLA), and polycaprolactone (PCL) have been investigated due to their suitable biocompatibility and mechanical properties [8,9]. However, the full-thickness circumferential esophageal replacement using tissue-engineered substitutes remains a challenge [10]. Different methods are currently being explored to develop esophagus substitutes using cells, polymer scaffolds, or a combination of both, according to the severity of lesions to be treated [10]. In these terms, electrospinning is an advantageous technique for manufacturing tubular nanofibrous polymer scaffolds made of fibers ranging from the submicron level to several nanometers in diameter in a high voltage electrostatic field [9,11]. Electrospun nanofibers have the advantage of mimicking the human extracellular matrix (ECM) and guaranteeing a cell’s suitable support for growing and proliferation, allowing excellent results in tissue regeneration [12,13]. Among the different cell types, mesenchymal stem cells (MSCs) are of great interest to both clinicians and researchers for their great potential to enhance tissue regeneration [14]. Moreover, MSCs secrete a variety of growth factors, cytokines, and exosomes that promote neovascularization and regulate immune cell activities, and they exhibit chemo-attractive and anti-inflammatory properties [15]. The well-documented self-renewal and differentiation capacities make the use of MSC a promising and valuable approach for electrospun scaffolds cellularization for TE purposes [16,17]. In a recent study published by La Francesca et al., full-thickness circumferential esophageal regeneration was achieved with synthetic polyurethane electrospun grafts seeded with autologous adipose-derived MSCs using a porcine model. The polyurethane graft was not integrated into the newly synthetized tissue and was removed after 3 weeks, showing a gradual structural regrowth of endogenous esophageal tissue [16]. Tan et al. demonstrated in a dog model that bone marrow MSCs on an SIS scaffold (Surgisis^®^; Cook Biotech Inc., West Lafayette, IN, USA) can promote re-epithelialization, revascularization, and muscular regeneration [18]. Moreover, it was demonstrated that autologous MSCs accelerated esophagus mature re-epithelialization and initiation of muscle cell colonization in a minipigs model. For full-thickness esophageal replacement, the use of cellularized biocompatible matrices has shown the best outcome in translational models [19].

The aim of this work was the development of a full-thickness esophageal engineered scaffold. Electrospinning technique was used to obtain tubular polymeric electrospun scaffolds made from Poly-L-lactide-co-poly-ε-caprolactone (PLA-PCL) 70:30 and 85:15 ratios. Previous studies of the research group demonstrated that PLA-PCL have good properties of biocompatibility and biodegradability and represent a good support for esophageal tissue regeneration [11,17]. Moreover, the combination of PLA-PCL with different ratios can modulate the scaffold permeability and mechanical properties. Electrospun tubular scaffolds, either cylindrical or asymmetrical, were characterized for their structural features concerning fiber dimension and orientation, porosity, wettability, degradation, and mechanical properties. Mesenchymal stem cells obtained from porcine bone marrow (p-MSCs) were expanded and used to cellularize the tubular scaffolds. Biological characterization was performed to select a more suitable cell seeding method and to determine cell viability and distribution on the scaffolds surface. Eventually, an in vivo porcine model was set up, and implantation of the selected most suitable tubular scaffold was investigated.

## 2. Materials and Methods

### 2.1. Scaffold Preparation

Copolymers Poly-L-lactide-co-poly-ε-caprolactone (PLA-PCL) 70:30 ratio (Resomer LC 703 S—Mw 160,000 Da-Evonik Nutrition & Care GmbH, Evonik Industries AG Rellinghauser Straße 1–11, 45128 Essen, Germany) and 85:15 ratio (PURASORB PLC 8516—Corbion, Piet Heinkade 127 1019 GM Amsterdam, The Netherlands) were chosen to prepare polymeric tubular scaffolds. 

Polymeric solutions were prepared at 20% *w*/*v* in Methylene Chloride (MC) and *N*,*N*-Dimethylformamide (DMF) solvent blend 70:30 ratio. MC was chosen due to its good solvent capacity towards PLA-PCL copolymer and for its low boiling point (40 °C), which allows fast solvent evaporation during the electrospinning process. Instead, DMF has a high boiling point (150 °C) but was selected due to its high dielectric constant (ε = 36.70), promoting fiber stretching through an electric field [20].

An electrospinning apparatus (NANON-01A) equipped with dehumidifier (MEEC instruments, MP, Pioltello, Italy) was used to produce electrospun tubular scaffolds.

Two different types of rotating collectors were used for fiber deposition: a cylindrical mandrel and an asymmetrical mandrel. The cylindrical mandrel had a diameter of 10 mm, while the asymmetrical one had one 8 mm diameter portion and a second 10 mm diameter portion. Asymmetrical geometry was selected in order to suit surgical implantation technique. Mandrels were coated with aluminum foil to increase conductivity and facilitated scaffold recovery from themselves.

Electrospinning process parameters were set up in a previous work as follows: voltage: 30 kV, flow-rate: 0.5 mL/h, speed of rotating mandrel: 1500 rpm, needle-collector distance: 15 cm, spinneret speed: 70 mm/sec, spinneret width: 70 mm, temperature: 25 ± 3 °C, and relative humidity (RH%): 30 ± 4% [11].

PLA-PCL 85:15, 20% *w*/*v* solution was electrospun simultaneously with PLA-PCL 70:30, 20% *w*/*v* solution using two different syringes for the first 10 min; for the remaining spinning time (20 min), only PLA-PCL 70:30, 20% *w*/*v* solution was electrospun in such a way to obtain double-layer scaffolds where the inner layer was richer in PLA because it was composed of PLA-PCL 85:15 and PL–PCL 70:30. The electrospinning process and fiber collection are influenced by the charge density (*σ*) on the mandrel surface: a higher charge density allows better jet stretching, resulting in more aligned polymeric fibers [21]. This can be explained introducing Gauss’ theorem, according to which the electric field *E* is directly proportional to charge density *σ* on mandrel surface as demonstrated in Equation (1):

(1) $$E\;=\frac{\sigma }{2\times \varepsilon_{0}}\hat{n}$$

where *E* is the electric field (Newton/Coulomb), *σ* is charge density (C/m^2^), *ε*_0_ is vacuum dielectric constant [8.9 × 10^−12^ C^2^/(N*m^2^)], and $\hat{n}$ is the versor perpendicular to mandrel surface.

If mandrel surface is not perpendicular to the electric field lines, the angle *α* at which the electric field lines cross the surface must be taken into account. Changing the mandrel geometry leads to change surface charge density.

**Figure 1:**
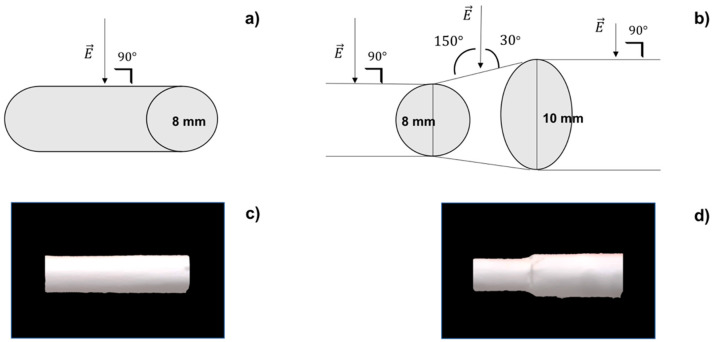
(**a**) Cylindrical mandrel surface perpendicular to electric field; (**b**) asymmetrical mandrel junction not perpendicular to electric field; (**c**) polymeric cylindrical scaffold; (**d**) polymeric asymmetrical scaffold.

The surface of cylindrical mandrel is always perpendicular to the jet (Figure 1a), so charge density (*σ*) can be calculated as follows from Equation (2):

(2) $$\sigma =E\times 2\times \varepsilon_{0}$$

where *E* = 2.5 × 10^6^ V/m is the electric field intensity of the electrospinning set up for 15 cm needle-collector distance with an applied voltage of 30 kV [22].

Instead, in the case of the asymmetrical mandrel, the junction between the two portions of different diameters can be considered similar to a truncated cone, where the jet is no longer perpendicular to the surface (Figure 1b) but will strike it forming an angle *α* of 30° or 150° (following role of complementary angles). Equation (3) can be used to calculate surface charge density on asymmetrical junction:

(3) $$\sigma =\frac{E\times 2\times \varepsilon_{0}}{cos\alpha }\;\;\;\;\;\;\;\;\;\;\;\;\;$$

### 2.2. Scaffold In Vitro Characterization

The electrospun tubular scaffolds with different geometries (cylindrical and asymmetrical) were characterized for their morphology, permeability, degradation, mechanical properties, and biological behavior. The results obtained were compared to select more suitable scaffold geometry for esophageal application.

#### 2.2.1. Scanning Electron Microscopy

SEM analysis was conducted on polymeric scaffolds to evaluate the following parameters: fiber diameters (nm), matrix pore areas (nm^2^) and porosity % per unit surface area, and fiber orientation (°). Zeiss EVO MA10 apparatus (Carl Zeiss, Oberkochen, Germany) was used. Analyses were performed at the following magnifications: 1.00 kX, 2.50 kX, and 300 kX. Both cylindrical and asymmetrical scaffolds were analyzed. The resulting images were processed by ImageJ software (open-source image processing program designed for scientific multidimensional images—National Institutes of Health, Bethesda, MD, USA) using DiameterJ (DiameterJ v1.018 ImageJ software, USA) and OrientationJ (OrientationJ_.jar ImageJ software, USA) plugins in order to achieve data of fiber size, porosity, and orientation [23].

#### 2.2.2. Permeability

Both cylindrical and asymmetrical scaffolds were characterized in vitro in terms of permeability towards 180 Da molecular weight molecules. Here, 180 Da was chosen to mimic the molecular weight of simple nutrients such as oxygen and carbon dioxide, which are essential for the life of cells; in particular, glucose was used to assess the permeability of 180 Da [24]. The potassium Ferrocyanate method was used for quantitative glucose determination [25]. This method is based on redox reaction of potassium ferrocyanate (329.24 g/mol), which reacts by oxidizing glucose to form gluconic acid.

The glucose concentration is inversely proportional to the concentration of potassium ferrocyanate left in solution after oxidizing reaction. A glucose calibration curve in the range 0.015–0.5 mg/mL was drawn as follows. A 1 mL of potassium ferrocyanate solution (K_3_Fe(CN)_6_ 0.015 M + Na_2_CO_3_ 0.5 M) was added to 1 mL of each glucose solution standard concentration. The reaction mixtures were heated for 15 min at 70–80 °C. Distilled water was added to each concentrations up to a total volume of 10 mL, and the resulting solutions were diluted 1:2 with distilled water and analyzed with a UV-vis spectrophotometer (6705 UV/Vis Spectrophotometer—Single cell holder JENWAY) at 420 nm wavelength.

The absorbances (Abs) obtained relates to the amount of not reacted potassium ferrocyanate. The calibration curve that correlates Abs to glucose concentration (mg/mL) was derived from glucose: K_3_Fe(CN)_6_ (1:2) stoichiometric ratio.

The permeability test was performed on cylindrical and asymmetrical scaffolds as follows. First, 1 mL of 10 mg/mL glucose solution was introduced into each scaffold that was closed with two clips. The scaffold was then introduced into a falcon tube containing 39 mL of PBS (pH = 7.4) and immersed in a thermostated bath (37 °C) under magnetic stirring (700 rpm).

Then, 1 mL samples were taken successively from the external medium; the withdrawal times were: 15′, 30′, 1 h, 2 h, 4 h, 6 h, and 24 h. After each sampling, the external medium was reconstituted, adding 1 mL of fresh PBS to keep sink conditions.

The 1 mL obtained at different timing points were added to 1 mL of potassium ferrocyanate solution (K_3_Fe(CN)_6_ 0.015 M + Na_2_CO_3_ 0.5 M). The reaction mixtures were heated for 15 min at 70–80 °C. Distilled water was added to each sample, up to a total volume of 10 mL, and the resulting solution was diluted 1:2 with distilled water and analyzed with a UV–vis spectrophotometer (6705 UV/Vis. Spectrophotometer—Single cell holder JENWAY, Cole-Parmer, Beacon Road, Stone, Staffordshire, ST15 OSA, UK) at 420 nm wavelength.

#### 2.2.3. Wettability

Wettability is a physical parameter that expresses the affinity of a liquid to a solid. It is a relative characteristic that needs to be referred to a specific solid, related to a specific liquid at a defined temperature. This parameter can be calculated measuring the contact angle (θ), that is, the angle included between the solid surface and the tangent to the wetting agent after a liquid drop is put on the solid surface. Perfect wettability corresponds to θ = 0, high wettability to 0 < θ < 90°; low wettability to 90 < θ < 180° and null wettability to θ >180°.

Contact angle meter DMe-211Plus was used to analyze the contact angle of the polymeric electrospun scaffolds. Wettability was measured 1 min after liquid drop-solid contact at room temperature, using a drop volume of 9 μL. Water and artificial saliva have been used as liquids for contact angle determination.

Wettability values were estimated for internal scaffold layer (made by a blend of PLA-PCL 70:30 and PLA-PCL 85:15) and external layer (made of PLA-PCL 70:30) of tubular scaffolds. Analyses were performed in triplicate for each sample considered, and data were reported as average ± standard deviation.

#### 2.2.4. In Vitro Degradation Test

The aim of the test was to evaluate in vitro degradation behavior of scaffolds in simulated physiologic conditions. The monitored parameters were pH of incubation medium, scaffold mass loss (ML) % and artificial saliva uptake (ASU) %, scaffold molecular weight (Mw), molecular number (Mn), and polydispersity index (PI).

In vitro degradation test was fulfilled in triplicate either for cylindrical or asymmetrical scaffolds. The test was performed for 14 days in artificial saliva in order to mimic the physiologic conditions in which the scaffold will be found after implantation in the pig’s esophagus. The artificial saliva was prepared according to the formulation developed by Shellis in 1978 containing not only ions, vitamins, and aminoacids but also growth factors, mucin, and enzyme (Table S1 in supplementary data) [26].

Each scaffold was weighed and inserted in a falcon tube containing 10 mL of artificial saliva. The samples were introduced in a thermostated bath at 37 °C.

- Control of Artificial Saliva pH

Measures of pH were carried out with a pH meter (pH meter 827 pH lab Metrohom, Via Giuseppe Di Vittorio, 5 21040 Origgio (VA), Italy). The control of artificial saliva pH was carried out every two days for two weeks.

- Determination of ASU%

The original mass (*M*_0_) of each scaffold was determined before starting in vitro degradation test by weighing scaffolds using analytical balance (mod AG245, Mettler-Toledo S.p.A., Via Anna Maria Mozzoni 2/1, 20152, Milano, Italy).

Scaffolds were extracted from artificial saliva every 24 h for 14 days; the exceedance of medium was removed by absorption with paper towel and final weight (Mt) was determined gravimetrically. The percentage of artificial saliva uptake is defined by Equation (4):

(4) $$ASU\;\%=\frac{M_{t}-M_{0}}{M_{0}}\times 100$$

where *M_t_* is the weight of scaffold at generic timing *t*, and *M*_0_ is the initial weight of scaffold.

- Determination of ML%

Mass loss % of scaffolds incubated at 37 °C in artificial saliva was measured at the 7th and 14th days.

Each sample was removed from artificial saliva medium and washed with distilled water to eliminate saliva residues that can adhere to the patch surface. Then, the sample was frozen for 5 h at −25 °C and then freeze-dried at −48 °C, 0.4 mbar for 12 h (Lio 5P) in order to remove traces of water. The freeze-dried scaffold was then weighted. The percentage of mass loss was calculated with the application of Equation (5).

(5) $$ML\;\%=\frac{M_{0}-M_{x}}{M_{0}}\times 100.$$

where *M_x_* is the weight of freeze-dried patch at various timing, and *M*_0_ is the weight of freeze-dried patch at time 0.

- Determination of Molecular Weight (MW), Molecular Number (Mn), and Polidispersity Index (PI)

Agilent Technologies 1260 Infinity GPC apparatus equipped with a precolumn (Agilent GPC/SEC Guard Columns) and three Ultrastyragel columns connected together in series (7.7 × 250 mm, each with different pore diameters 104 Å, 103 Å, and 500 Å), an IR detector (Agilent Technologies 1260 Infinity) and software to handle data relative to Mn, Mw, and PI (OpenLab and Cirrus), was used.

Before sample injection, a calibration curve of styrene standards at defined molecular weight (8100 Da, 70,950 Da, 105,000 Da, 143,400 Da, 315,500 Da) in THF was achieved.

The same cylindrical and asymmetrical scaffolds that underwent ML determination were solubilized in THF at 1 mg/mL concentration. The solutions obtained were filtered with a Fluoropore 0.45 μm (Millipore, Burlington, MA, USA) filter and injected in GPC apparatus with THF as mobile phase at constant flow rate of 1 mL/min.

#### 2.2.5. Scaffolds Cellularization

Porcine bone marrow mesenchymal stem cells (p-MSCs) were used for scaffold cellularization. Pigs kept at the animal enclosure of the University of Pavia, after approval for experimental use by the Ministry of Health, underwent general anesthesia, and 20–40 mL of bone marrow aspirate was taken from their upper iliac crests. The samples were diluted and subjected to density gradient separation.

Mononuclear cells (MNCs), recovered from the Lymphoprep interface, were plated in uncoated polystyrene flasks for cell culture (Corning Costar, Celbio, Milan, Italy) at a concentration of 160,000/cm^2^, and proliferation was ensured by the addition of DMEM-LG (low glucose) (Gibco Invitrogen, Paisley, UK) with gentamicin 50 μg/mL (Gibco Invitrogen) and 10% *v*/*v* fetal bovine serum (FBS; Mesenchymal Stem Cell stimulatory Supplements, StemCell Technologies, Vancouver, BC, Canada).

Flasks were maintained in culture at 37 °C in a humidified atmosphere enriched with 5% CO_2_. After 48 h, non-adherent cells were removed by replacing the culture medium with fresh medium.

When p-MSCs reached ≥80% confluence, they were treated with trypsin (Trypsin-EDTA; Sigma-Aldrich, Milan, Italy), counted and plated at a density of 4000 cells/cm^2^, and expanded to passage P4.

It should be noted that each treatment performed on the cell cultures was carried out under a vertical laminar flow hood (Ergosafe, Space 2, PBI International, Milan, Italy) with sterile disposable or autoclaved instruments and materials. p-MSCs characterization was reported in a previous study [17]. The tubular scaffolds underwent sterilization by ionizing irradiation before cell seeding, according to the EMA “Guideline on the sterilization of the medicinal product, active substance, excipient and primary container” and following a protocol previously set up and discussed by the authors [27]. Each sample was located in aluminum pounces (polyamide/aluminum/polyester peel for γ-sterilization, VWR) and hermetically closed; the irradiation was carried on at Laboratorio di Energia Nucleare Applicata (LENA) University of Pavia, Italy, using Cobalt-60 as γ-rays source at a total irradiation dose of 25 kGy.

Tubular scaffold cellularization with p-MSCs was performed as explained below, following two methods on purpose set up by the authors in order to adapt it to the scaffold geometry and optimize cell attachment and proliferation.

(1). Vertical seeding and incubation into one side closed scaffold: the scaffold was closed at one end with a clip and placed vertically in a falcon tube. The operator seeded the cells suspension inside the scaffold from the opened scaffold top. After 48 h, the scaffold was horizontally placed in 6-multiwell, and the clip was removed.
(2). Horizontal seeding and incubation into two-side closed scaffold: the scaffold was closed at one end with a clip. The operator seeded the cells suspension inside the scaffold and then closed the other end with a second clip. The scaffold was placed horizontally and turned over after 2 h.

Each of these techniques was carried out on cylindrical and asymmetrical scaffolds in triplicate. Each patch was seeded with 1,000,000 p-MSCs. 

#### 2.2.6. Cell Viability and Staining

The p-MSCs scaffolds underwent 3-(4,5-dymethiltiazol-2-y)-2,5 diphenyltetrazolium bromide (MTT) test in order to evaluate cellular viability. After 7 and 14 days incubation, the culture medium was removed, and scaffolds were washed with PBS. Then, 300 μL of a 5 mg/mL solution of MTT were added on each scaffold sample, and fresh PBS was added in order to guarantee the total immersion of the samples. After 2 h and 30 min incubation at 37 °C and 5% of CO_2_, the medium was removed, paying attention not to suck precipitated formazan salts.

The scaffold samples were transferred from the multiwell plate to a vial and solubilized with tetrahydrofuran (THF) under magnetic stirring for 1 h in order to completely dissolve the polymeric matrix and lyse cellular membranes of cells adhered on the polymer. The lysate cellular membranes released formazan crystals that solubilized, obtaining a violet solution. p-MSCs treated with MTT and solubilized in DMSO were used as a positive control.

Polymeric solutions obtained by dissolution of cellularized scaffolds and positive control were spectrophotometrically analyzed at 570 nm wavelength in a quartz cell (6705 UV/Vis Spectrophotometer—Single cell holder JENWAY). THF is used as a blank in all the readings. DAPI (4′,6-diamidino-2-phenylindole) is a fluorescent stain that binds strongly to adenine–thymine-rich regions in DNA in order to highlight the nuclei of the cells and detect cell on scaffolds. At prefixed incubation time points (7 and 14 days), scaffolds were washed with PBS, and glutaraldehyde 0.4% *v*/*v* was added and left for 10 min in order to fix the cells. Scaffolds were washed one more time with PBS, and TRITON X 0.1% *v*/*v* was added and left for 10 min in order to permeabilize the cell membranes. After a following washing step with PBS, scaffolds were treated with DAPI 300 nM solution overnight at 4 °C. The samples were analyzed with fluorescence microscope (Leica DM IL LED with ebq 50 ac-L), and images obtained were processed by ImageJ software. SEM analysis was carried out to investigate cell orientation and infiltration into the electrospun nanofibrous matrices. At prefixed time points (7 and 14 days), the scaffolds were washed with PBS. Dehydration was carried out with ethanol (EtOH) solution at increasing concentrations (30% *v*/*v*, 70% *v*/*v*, 80% *v*/*v*, and 100% *v*/*v*); each passage involved 10 min of contact with ethanol. The samples were washed with a 50:50 mixture of dry EtOH 100% and hexamethyldisilazane (HDMS) for 20 min. Solvent excess was removed, and the samples were left under a laminar hood for 2 h; the scaffolds were made conductive thanks to deposition on their surface of a thin layer of gold in an Argon atmosphere before SEM analysis. SEM images were further processed by ImageJ software in order to characterize cellularized scaffolds.

#### 2.2.7. Mechanical Properties

Knowledge of scaffold mechanical properties is important in order to understand if they are able to withstand the physiological movements of esophageal tract.

The mechanical properties of explanted pig esophagus were evaluated and taken as standard. The mechanical properties of the cylindrical and asymmetrical scaffolds were analyzed in order to assess which of these scaffolds have properties most similar to those of the explanted pig esophagus. The mechanical properties were monitored along in vitro degradation test in artificial saliva. Cylindrical and asymmetrical scaffolds were analyzed at t_0_ (before in vitro degradation test), t_7_ (after 7 days incubation in artificial saliva), and t_14_ (after 7 days incubation in artificial saliva). Mechanical properties were also evaluated for engineered scaffolds after 7 and 14 days of cellularization. The pig esophagus and scaffolds were cut in a “dog bone” shape of width equal to 0.4 cm and length equal to 2.5 cm by the ISO standardized die-cutting machine (ISO 17025:2017). This “dog-bone” shape is a standardized one and makes results comparable, as it allows force to always be applied at the same point.

The samples were subjected to the tensile test at room temperature using a tensiometer (Mark-10 Tensile Testing, MARK-TEN, Copiague, NY, USA). The samples were placed between the grippers of the tensile stress tester; the grippers moved away with a constant and predetermined speed. The loading velocity set up was 50 mm/min, used to characterize human esophagus, according to the literature [28].

### 2.3. Scaffold In Vivo Implantation: Surgical Procedure

All animal experiments were conducted in accordance with the ARRIVE guidelines and EU Directive 2010/63/EU for animal experiments [29,30]. Animal experimentation was authorized by ministerial approval (Italian Health Ministry) with the following project code: n° 842/2017-PR del 26/10/2017.

In vivo experiments were performed on 9–12 month-old female pigs (landrace/large white), weighing 27.5 ± 5.44 kg. Two groups of 2 animals were used for implantation of the scaffolds, respectively, with absorbable continuous suture and detached stitch suture.

Animals were fasted for 12 h before surgery. Under general anesthesia with intravenous thiopental sodium and intubation, they were maintained by means of inhalation of isoflurane and O_2_. Cephalosporin dissolved in 50 mL of physiological saline was administered intravenously immediately before the incision. The animals were immobilized in a left lateral position. Their hair was clipped, chlorhexidine or povidone iodine was used to clean the skin, and each pig was covered with sterile drapes. A standard right thoracotomy at the level of the 4th intercostal space on each animal was performed, and the thoracic cavity was entered. About 5 cm of thoracic esophagus was exposed by right thoracotomy. A full-thickness circumferential segment of the esophagus (2.5 cm long, located in the mid thoracic region, posterior to the right lung hilum) was resected. In the first group (*n* = 2), the asymmetrical engineered scaffold was implanted using 4-0 polydioxanone (PDS) absorbable continuous suture with anastomosis to the proximal and distal esophagus. In the second group (*n* = 2), instead, detached stitch sutures with 4-0 polydioxanone (PDS) on both sides at the excision site were performed. Asymmetrical scaffold, due to its peculiar geometry, favored surgical operation, allowing us to perform an underlay upper suture and an overlay lower suture able to guarantee continuity between the scaffold and the esophageal tissue.

Postoperative monitoring was performed daily throughout the animal survival, assessing respiratory and surgical complications. Within each group, sacrifice of animal was planned at 7 days after implantation. The whole esophagus was harvested by a new surgery. Following euthanasia by an intravenous ketamine hydrochloride overdose, post-mortem esophagectomies were performed via thoracotomy. Animal weights were assessed before the first and second operations. Excised esophageal segments were evaluated and described. All results were compared in the patch, surgical wound, and normal esophageal tissue.

### 2.4. Statistical Analysis

The results collected in this experimental study were presented as the mean and standard deviation (SD). A statistical analysis tool was performed in Microsoft Excel (Office 365 Microsoft, Redmond, WA, USA) using analysis of variance (ANOVA). A *p*-value less than 0.05 (*p* < 0.05) is considered statistically significant, while a *p*-value higher than 0.05 (*p* > 0.05) is not statistically significant and indicates strong evidence for the null hypothesis. All experiments were carried out in triplicate three time (*N* = 9) unless otherwise stated. Data are presented as average ± SD, * *p* < 0.05; ** 0.1 > *p* > 0.05.

## 3. Results

### 3.1. Scaffold Preparation 

Electrospun tubular scaffolds were obtained using pre-optimized process parameters [11]. The same process parameters were used to collect cylindrical and asymmetrical polymeric scaffolds. Using the cylindrical mandrel, a scaffold of 10 mm diameter was obtained (Figure 1c), while with asymmetrical mandrel, a scaffold with a smaller diameter of 8 mm (d_1_) and a larger dimeter of 10 mm (d_2_) connected by a truncated cone junction was obtained (Figure 1d).

Cylindrical and asymmetrical mandrel surfaces influenced fiber collection. Changing the mandrel geometry leads to change charge density (*σ*) that influenced fiber deposition and orientation. Applying inverse forms of Gauss’ equation (Equation (2) for cylindrical scaffold and Equation (3) for asymmetrical scaffold), the values of charge density for cylindrical and asymmetrical mandrels were calculated. The junction part of the asymmetrical mandrel showed higher values of charge density (0.13 C/m^2^) compared to the cylindrical mandrel (0.11 C/m^2^). Higher charge density values allow to obtain more aligned fibers. Morphological characterization by SEM confirmed that theoretical evaluation.

### 3.2. Scaffold In Vitro Characterization

#### 3.2.1. Scanning Electron Microscopy

**Figure 2:**
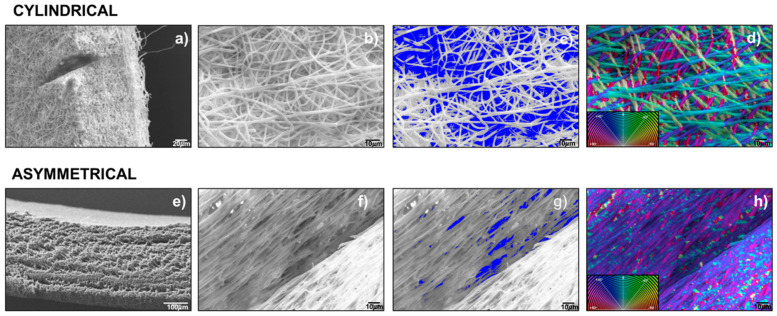
Images of cylindrical scaffold: (**a**) SEM-scaffold thickness, (**b**) SEM- scaffold inner surface, (**c**) Imagej segmentation of SEM image, (**d**) Imagej orientation of SEM image (**a**). Images of asymmetrical scaffold: (**e**) SEM-8 mm diameter scaffold thickness, (**f**) scaffold inner surface, (**g**) Imagej segmentation of SEM image, (**h**) Imagej orientation of SEM image (**f**).

Scaffold images obtained by SEM were processed with ImageJ software program and the data of fiber diameter (μm ± SD), mean pore area (μm^2^ ± SD), and porosity % were generated as output and reported in Table 1. Cylindrical scaffolds were characterized for thickness, fiber diameter, fiber porosity %, and orientation. The cylindrical scaffold showed a thickness of 370 ± 0.03 μm, a fiber diameter of 1.16 ± 0.27 μm, a mean pore area of 22 ± 17 μm^2^, and a fiber porosity % on a normalized area of 8.27 ± 2.7%. Fiber orientation showed a random fiber dispersion (Figure 2d). Asymmetrical scaffold characterization of the three portions of scaffold (8 mm portion, junction, and 10 mm portion) was performed by SEM. The 8 mm portion shows a thickness of 329 ± 7.4 μm, a fiber diameter of 0.95 ± 0.1 μm, a mean pore area of 19 ± 8.4 μm^2^, and a fiber porosity % on normalized area of 3.78 ± 0.42%. Characterization of a 10 mm portion showed a thickness of 297 ± 11.9 μm, a fiber diameter of 1.1 ± 0.5 μm, a mean pore area of 19 ± 14.2 μm^2^, and a fiber porosity % on normalized area of 8.55 ± 0.64%.

The 10 mm portion of asymmetrical scaffold shows a lower thickness compared to 8 mm portion; this is probably due to its higher surface area. The junction showed a width of 1025 ± 59.3 μm, a fiber diameter of 2.07 ± 0.37 μm, a mean pore area of 67 ± 64.1 μm^2^, and a fiber porosity % on normalized area of 0.17 ± 0.3%. Due to the higher value of charge density of this portion of the mandrel, fibers showed a more defined alignment. This higher alignment induces a lower porosity in the fiber network compared to random orientation. Moreover, mean fiber diameter is higher compared to other portions of the scaffold, because the alignment makes it difficult to be measured by software. Representative SEM images of asymmetrical scaffold junction are reported and compared to that of the cylindrical scaffold (Figure 2).

SEM images of 8 mm and 10 mm portions of electrospun asymmetrical scaffold (Supplementary data—Figure S2). Confirmed greater orientation in the junction portion (higher σ value) compared to two other portions (lower σ values) that have surfaces perpendicular to the jet.

#### 3.2.2. Permeability

**Figure 3:**
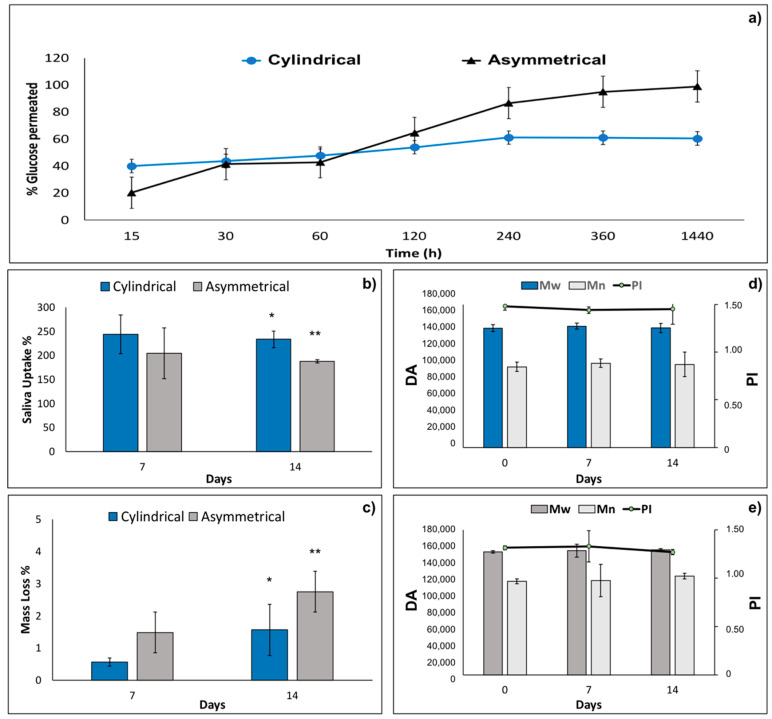
(**a**) Results 180 Da permeability test performed on tubular and asymmetrical scaffolds at 37 °C in PBS (pH = 7.4). (**b**) Artificial saliva uptake %, (**c**) mass loss % of cylindrical and asymmetrical scaffolds after 7 and 14 days in incubation in artificial saliva at 37 °C. Molecular weight (Mw), molecular number (Mn), and polydispersity index (PI) variations after 7 days and 14 days in incubation with artificial saliva for (**d**) cylindrical scaffold and (**e**) and asymmetrical scaffold. Data are presented as mean ± SD, * *p* < 0.05; ** 0.1 > *p* > 0.05.

Results of 180 Da permeability test performed on cylindrical and asymmetrical scaffolds are reported in Figure 3a. After 1440 min (24 h), incubation of the asymmetrical scaffold is more permeable (98.94 ± 16.00%) than the cylindrical one (60.41 ± 1.77%). This behavior is probably due to the smaller thickness of the two portions of asymmetrical scaffold and the higher pores area of junction section (Table 1).

#### 3.2.3. Wettability

Compatible with the hydrophobic nature of copolymers PLA-PCL wettability is low (90 < θ < 180°) for both liquids analyzed (Table 2).

Both in water and artificial saliva, the internal layer showed more hydrophilic behavior than did the outer layer. The result is consistent with the higher hydrophobicity of PCL compared to PLA. Using artificial saliva, the values of contact angle are always lower than in water due to the lower surface tension of artificial saliva (63.95 ± 1.44 dyne/cm) compared to water (72.8 ± 0.9 dyne/cm).

#### 3.2.4. In Vitro Degradation Test

The values of artificial saliva pH incubated with cylindrical and asymmetrical scaffolds kept in the pH range of 6.37–6.98 along the 14 days of in vitro degradation test. The results of artificial saliva uptake % for cylindrical and asymmetrical scaffolds are reported in Figure 3b. Cylindrical scaffolds showed higher absorption of saliva at both 7 days and 14 days (244.1% and 233.7%, respectively) compared to the asymmetrical ones (204.8% and 187.9%). This behavior is probably due to the higher surface area of the cylindrical scaffolds (18.85 cm^2^) compared to the asymmetrical ones (16.96 cm^2^). Mass loss % of cylindrical and asymmetrical scaffolds were evaluated by gravimetric method after 7 and 14 days’ incubation in artificial saliva, and the results are reported in Figure 3c. The asymmetrical scaffolds show higher mass loss % after 7 and 14 days (1.5 ± 1.0% and 2.7 ± 1.1%, respectively) compared to the cylindrical ones (0.6 ± 0.1% and 1.6 ± 0.7%). Moreover, mass loss % reaches very low values after 14 days of incubation for both scaffolds analyzed, demonstrating that the scaffolds are stable after 14 days’ incubation time in simulated physiological conditions. Indeed, macroscopic morphology of the scaffolds remained constant after 14 days incubation.

Molecular weight (Mw), molecular number (Mn), and polydispersity index (PI) were evaluated both for cylindrical and asymmetrical scaffolds after 14 days’ incubation in artificial saliva. The results reported in Figure 3d,e, respectively, show that Mw, Mn, and PI of cylindrical and asymmetrical scaffolds are comparable and did not undergo any significant changes after 7 days and 14 days of incubation in artificial saliva. In conclusion, the electrospun polymeric scaffolds are stable for 14 days in simulated physiologic conditions.

#### 3.2.5. Cell Viability and Staining

**Figure 4:**
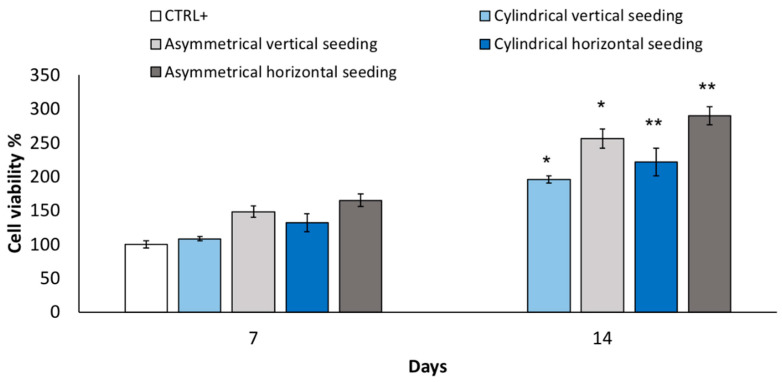
Cell viability % performed after 7 and 14 days’ incubation using vertical seeding method on cylindrical and asymmetrical scaffolds. Cell viability % performed after 7 and 14 days’ incubation using horizontal seeding method on cylindrical and asymmetrical scaffolds. Data are presented as mean ± SD, * *p* < 0.05; ** 0.1 > *p* > 0.05.

Cell viability % from the vertical seeding method was evaluated after 7 and 14 days for cylindrical and asymmetrical scaffolds. The results reported in Figure 4 show that asymmetrical scaffolds show a higher cell viability % (148 ± 8.6%) compared to CTRL+ and cylindrical scaffolds (108 ± 2.9%) after 7 days’ incubation. After 14 days’ incubation, a significant increase in cell proliferation is highlighted for both scaffolds, and the asymmetrical scaffolds confirmed the highest value of cell viability (256 ± 14%), either compared to CTRL+ and cylindrical scaffolds (195.7 ± 5.0%), respectively. In Figure 4, data of cell viability % obtained from horizontal seeding method are also reported. Cell viability % was evaluated after 7 and 14 days’ incubation for cylindrical and asymmetrical scaffolds. Compared to the control at 7 days, the asymmetrical patch shows an increase in cell viability % (164 ± 9.1%). After 14 days of incubation, the cell number duplicated corresponding to an increase of 289 ± 13% in cell viability % compared to 7 days’ CTRL+.

**Figure 5:**
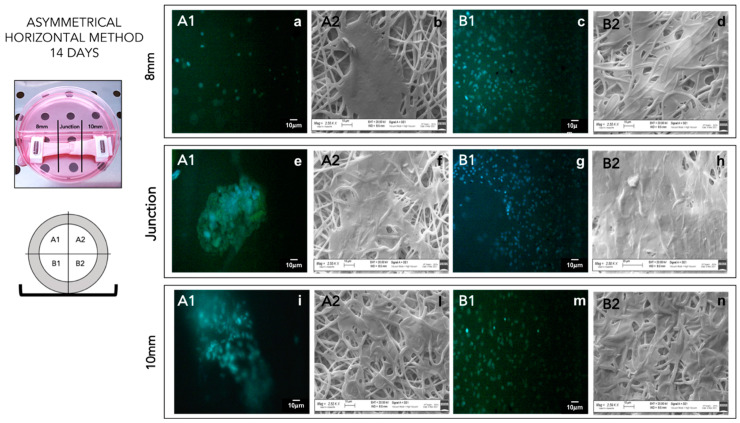
DAPI staining (A1–B1) of asymmetrical patch cellularized by horizontal seeding method after 14 days of incubation. SEM analysis (A2–B2) of asymmetrical patch cellularized by horizontal seeding method after 14 days of incubation. (**a**) 8 mm A1 portion DAPI staining; (**b**) 8 mm A2 portion SEM image; (**c**) 8 mm B1 portion DAPI staining; (**d**) 8 mm B2 portion SEM image; (**e**) junction A1 portion DAPI staining; (**f**) junction A2 portion SEM image; (**g**) junction B1 portion DAPI staining; (**h**) junction B2 portion SEM image; (**i**) 10 mm A1 portion DAPI staining; (**l**) 10 mm A2 portion SEM image; (**m**) 10 mm B1 portion DAPI staining; (**n**) 10 mm B2 portion SEM image.

In conclusion, the asymmetrical scaffolds cellularized with p-MSCs, using the horizontal seeding method, better promoted cell attachment and proliferation. Asymmetrical scaffolds seeded with horizontal method showed more uniform cell distribution on the whole scaffold surface of sections B in contact with the multi-well bottom. After 14 days’ incubation, a partial coverage of upper scaffold surface A was also achieved (Figure 5). Images of an asymmetrical scaffold seeded with the vertical seeding method after 14 days’ incubation showed non-uniform cellularization, compatible with the seeding method, with an higher concentration of cells on the lower section (see Supplementary data—Figure S1). After 14 days’ incubation, in the middle part, it was also possible to notice a consistent number of cells greater than those detected after 7 days.

#### 3.2.6. Mechanical Properties

Preliminary mechanical characterization was performed on cylindrical and asymmetrical scaffolds after electrospinning process (t_0_). Data of Young’s modulus, yield point, ultimate tensile strength, fracture point and elongation at break % were achieved after analysis on tensiometer. Esophagus obtained from pig was used as reference control. During in vitro degradation test performed in artificial saliva, mechanical properties of cylindrical and asymmetrical scaffolds were measured after 7 and 14 days’ incubation and are compared to those obtained from t_0_ and pig esophagus (Table 3).

Young’s modulus of cylindrical scaffold slightly decreased during the degradation test in artificial saliva. Instead, asymmetric scaffold showed at t_0_ a Young’s modulus value lower than that of cylindrical ones and more similar to the value of pig esophagus (0.27 ± 0.01 MPa). However, during the degradation test, Young’s modulus of asymmetrical scaffold increases, moving away from esophagus physiological values. Increasing Young’s modulus determines an increased brittleness of the scaffold. The slight decrease in Young’s modulus observed for the cylindrical scaffold (from 0.97 ± 0.10 MPa at t_0_ to 0.73 ± 0.2 MPa after 14 days) was always in value ranges higher than those of pig’s esophagus. Instead, the asymmetric patch, during the degradation test, had increased values of Young’s modulus (from 0.39 ± 0.07 MPa at t_0_ to 0.45 ± 0.08 after 14 days), becoming more brittle. Confirmation is given by ultimate tensile strength and yield stress values obtained for cylindrical and asymmetrical scaffolds. In brittle materials, the ultimate tensile strength is close to or even lower than yield stress, whereas in ductile materials, the ultimate tensile strength is higher than yield stress. At t_0_, both cylindrical and asymmetric scaffolds showed values of ultimate tensile strength more than six times higher than yield stress. After 7 days in artificial saliva, the gap between ultimate tensile strength and yield point slightly decreases, and after 14 days, the gap reduction is evident and significant for both scaffolds, but it is more noticeable for cylindrical scaffolds. For these scaffolds, the gap between ultimate tensile strength yield stress reverses, and ultimate strength becomes lower than yield stress. Increases in brittleness are also evident, analyzing the reduction in fracture point. For the asymmetric scaffold, the reduction in fracture point is more evident compared to the cylindrical scaffold. At t_0_, the asymmetric scaffold has a fracture point of 6.13 ± 0.2 MPa, which decreases to 5.05 ± 0.06 MPa after 7 days and to 4.72 ± 0.05 MPa after 14 days. Instead, for the cylindrical scaffold, the values of fracture point remain more or less constant until 7 days (at t_0_ = 3.89 ± 0.2 MPa, at t_7_ = 3.92 ± 0.7 MPa), while an evident decrease was achieved after 14 days (t_14_ = 1.86 ± 0.05 MPa). The fracture point of cylindrical scaffolds after 14 days is not compatible with the values obtained from pig’s esophagus (3.17 ± 0.56 MPa). The increase in brittleness is also correlated to the elongation at break %. Increasing the incubation time in artificial saliva, scaffolds showed a reduction in elongation at break %: the asymmetric scaffold showed, at t_0_, an elongation at break % of 273.1 ± 28% that consistently decreased at 215 ± 11% and 196 ± 14% after 7 and 14 days of incubation, respectively.

The same trend is also evident for cylindrical scaffolds: at t_0_, the value of elongation at break % was 260 ± 33%, at t_7_, it was 131.8 ± 32%, and at t_14_, was 119 ± 25%. Already, after 7 days, elongation at break % of cylindrical scaffolds was not suitable to sustain values of pig’s esophagus. For this reason, subsequent biological and mechanical characterization were performed only on asymmetric scaffolds. An asymmetric scaffold seeded with cells using horizontal method after 7 days of incubation showed value of Young’s modulus similar (0.25 ± 0.09 MPa) to those obtained for pig esophagus (0.27 ± 0.01 MPa). Compared to the control in DMEM at 7 days that showed a Young’s modulus of 1.1 ± 0.03 MPa, engineered scaffolds showed more elastic behavior, probably due to the presence of cells that slowed down scaffold embrittlement. Engineered scaffold showed an improvement in elongation at break % (227 ± 32%) compared to the control in DMEM (121 ± 23%) and compatible with the value of pig’s esophagus elongation at break % (200 ± 18%).

However, after 14 days of incubation, both engineered scaffold and scaffold ctrl in DMEM demonstrated an increase in brittleness that can be confirmed by the reduction in gap between value of yield stress and ultimate tensile strength. The scaffold ctrl in DMEM showed a higher reduction in elongation at break % (61 ± 15%) compared to engineered scaffolds (177 ± 17%). Additionally, in this case, the presence of cells on the scaffold probably contributed to maintain greater elasticity compared to the control in DMEM. In addition, asymmetrical engineered scaffold after 7 and 14 days of incubation showed values of Young’s modulus, yield stress, ultimate tensile strength, fracture point, and elongation at break % more similar to those of the pig’s esophagus compared to asymmetrical control scaffold. For this reason, engineered scaffolds are demonstrated to be more suitable for implantation for circumferential esophageal substitution.

### 3.3. Scaffold In Vivo Implantation in Animal Model 

**Figure 6:**
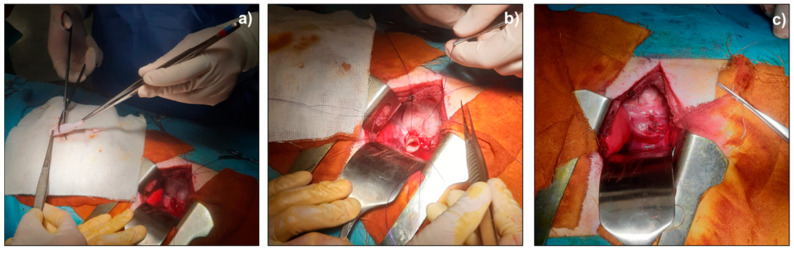
Surgical implantation of asymmetrical scaffold as pig’s esophagus substitute; (**a**) scaffold preparation before surgical implantation; (**b**) lower overlay suture; (**c**) final scaffold suture.

Asymmetrical scaffold was successfully implanted in pig animal model performing first of all a lower overlay suture and then completed tube connection was achieved with a upper overlay suture (Figure 6).

All four animals were alive at the end of the 7-day follow-up. In all cases, the early post-operative course was uneventful. The whole esophagus was harvested by a new surgery. The esophageal segments containing the patches were macroscopically evaluated immediately after the sacrifice. In the first animal group (continuous suture), we documented a partial detachment of the patch, with underlying inflammatory reaction. In the second group, we observed a good seal of the detached stitch suture without any leakage of the scaffold and no fistula formation; in particular, in each pig of the second group, there was no noticeable evidence of foreign body reaction or infection of the scaffold.

Preliminary in vivo results demonstrated that detached stitch suture is to be preferred to continuous suture to achieve better results in terms of animal welfare and tissue–scaffold integration.

## 4. Discussion

Esophageal surgery is associated with a mortality rate that can reach 13% at 90 days, and esophagectomy, which usually includes lymphadenectomy, remains the treatment of choice for esophageal malignancy. Gastrointestinal tract continuity is usually restored using gastric pull-up and, sometimes, the autologous interposition of colonic graft. The use of gastrointestinal segments to close the gap after esophageal resection has been the gold standard approach so far, despite postoperative complications that could lead to high morbidity. The most common postoperative complications are strictures, anastomotic leakage, mediastinitis, diarrhea, and reflux problems; pulmonary complications can arise in up to 38% of the patients [31]. For this reason, a significant level of research is being undertaken to achieve better results in esophagus reconstruction after resection, avoiding use of an autologous stomach or intestine conduit in favor of a bioengineered construct. Esophageal tissue engineering is a promising approach to create an esophageal substitute and improve clinical results in esophageal surgery, but it remains a challenging topic, even due to those difficulties arising from the immunogenicity and low biocompatibility of materials [5].

The strategies found in the literature for esophageal reconstruction mainly include these different approaches: (i) use of biologic material, (ii) use of polymer scaffolds, and (iii) combining polymer scaffolds to biologic material.

Previous studies have explored the use of non-absorbable or absorbable materials in esophageal repair; non-absorbable materials, such as plastic tubes of polyethylene terephthalate mesh or polytetrafluoroethylene sheet, have been used [32,33]. However, since this method involves permanent placement of a foreign material in the human body, it has been unsuccessful in clinical settings. Therefore, bioabsorbable materials should be used for human treatment. Natural absorbable materials, such as small intestinal submucosa, amniotic membrane, lyophilized dura mater, collagen matrix, and extracellular matrix with autologous muscle tissue, can be used. Nevertheless, these materials have not yet been used for clinical purposes due to poor regeneration of the muscular layer, cicatricial stenosis, and risk of infection. Aikawa et al. developed a bioabsorbable polymer patch able to repair defects in the esophageal wall in a porcine model without complications, and which induced the formation of a neo-esophageal wall identical to the native one in 12 weeks [34].

In this work, a polymer electrospun tubular scaffold is proposed, made from PLA-PCL, whose biocompatibility is well known and tested. The tubular scaffolds were manufactured by electrospinning in order to achieve nanofibrous matrices resembling an ECM structure. Moreover, in a previous work, the same authors demonstrated that interaction between PLA-PCL electrospun matrices and macrophages caused a modulation of the latter. A sensitive anti-inflammatory response was promoted by these polymeric electrospun PLA-PCL matrices after 3 days’ incubation with monocytes, which were used as a model to study the immune response capacity of monocytes and monocyte-derived macrophages [35].

Several experimental models have explored the combination and in vivo implantation of elements required for tissue remodeling toward a specific organ phenotype. Bone marrow-derived mesenchymal stromal cells (MSC) have been used in several clinical trials for different applications, including tissue regeneration. Although, traditionally, MSC regenerative capacity was associated with their presumptive plasticity, their therapeutic effects seem to be mostly due to their paracrine function through the secretion of a broad range of bioactive molecules that induce regeneration of damaged tissue cells. From this perspective, MSC can be used for esophageal tissue repopulation and reconstitution.

In the present work, the selected proposed tubular scaffold made from PLA-PCL was engineered with p-MSCs by static incubation in cell medium (DMEM). Mesenchimal stem cells are proposed by several authors for their advantages in esophageal reconstruction. For example, Takeoka and coll. [16] developed a scaffold-free structure with a mixture of cell types using 3D-bioprinting technology, and they found that mesenchymal stem cells tended to show greater strength and expansion on mechanical testing and highly expressed α-smooth muscle actin and vascular endothelial growth factor on immunohistochemistry. Another approach found in the literature is to use decellularized matrices that could be derived from decellularized porcine or rat esophagus and are re-populated with meso angioblasts and fibroblasts for the muscle layer or with human adipose-derived stem cells after dynamic culture in a bioreactor [36], whereas our strategy is to manufacture a polymer scaffold as support to be populated by cells and create an environment suitable for esophageal reconstruction. The advantage of our protocol is the speed and reproducibility of the scaffold manufacturing process by electrospinning compared with biological material such as decellularized matrices.

We thoroughly investigated the PLA-PCL tubular scaffold for its physico-chemical properties, including morphology related properties, and we showed an interesting correlation between scaffold asymmetrical shape and its p-MSCs cellularization promotion. Moreover, scaffold cellularization helped in keeping the scaffold mechanical properties suitable for esophageal application. The advantage of our scaffold hybrid composition (polymer combined to p-MSCs) resides in the good mechanical properties and patency that permit scaffold implantation without any additional support. Other authors such as La Francesca et al. and Jensen et al. investigated similar hybrid polymer scaffolds made from different polymers such as polyurethane and Polylactide-co-glycolide/polycaprolactone blends, respectively, demonstrating their interest in the topic [37]. We proposed a PLA-PCL copolymer that is particularly advantageous due to its mechanical properties and biodegradation rate. Moreover, another innovation of our study is in the proposed asymmetrical scaffold shape, which facilitates scaffold in vivo implantation.

## 5. Conclusions

The experimental work developed is addressed to an engineered full-thickness scaffold for esophageal tissue regeneration. Preliminary in vitro and in vivo characterization highlight the suitability of the PLA-PCL electrospun tubular scaffold as support able to promote and guarantee appropriate esophageal regeneration. The preliminary results collected after in vivo implantation in a porcine model are promising; further in vivo studies are in progress to statistically validate the data. The engineered scaffolds proposed will find practical application as combined advanced therapy medicinal products, according to European regulation 1394/2007, since they are made from a medical device (scaffold) combined with cells embedded herewith. This implies that the polymer scaffold should be an integral part of the medicinal product, but it should also be certified as medical device by an authorized notified body, according to current European regulation 745/2017. Therefore, a fully physico-chemical characterization, together with clinical trials, is required. The European Medicine Agency (EMA) warns against the use of unregulated cell-based therapies, which may be ineffective and increase the risk of serious adverse reactions. This brief comment highlights that several characterization steps are still needed to obtain a fully characterization of combined ATMPs [10,38]. Moreover, the in vivo preliminary results in this work documented the suitability of detached stitch suture with 4-0 polydioxanone (PDS) as the technique that guarantees the best continuity between the patch and the esophageal wall. In the following phases of the experiment, the aim is to confirm suitable interaction between tissue and scaffold performing deepen immunological and histological analysis.

## Figures and Tables

**Table 1:** Morphologic characterization of tubular scaffolds.

Tubular Scaffold Type	Portion Diameter (mm)	Scaffold Thickness (μm)	Fiber Diameter (μm)	Mean Pore Area (μm^2^)	Fiber Porosity % on Normalized Area (%)
Cylindrical	10 mm	370 ± 0.03	1.16 ± 0.27	22 ± 17	8.27 ± 2.7
Asymmetrical	10 mm	297 ± 11.9	1.10 ± 0.5	19 ± 14.2	8.55 ± 0.64
	8 mm	329 ± 7.4	0.95 ± 0.1	19 ± 8.4	3.78 ± 0.42
	10 mm/8 mm (junction)	-	2.07 ± 0.37	67 ± 64.1	0.17 ± 0.3

**Table 2:** Contact angle for inner and outer layer of tubular scaffolds in distilled water and artificial saliva.

Layer Composition	Contact Angel Distilled Water (*θ*_H_2_O_)	Contact Angle Artificial Saliva (*θ*_AS_)
PLA-PCL 70:30 + PLA-PCL 85:15 (inner)	105.1 ± 1.3	92.4 ± 0.36
PLA-PCL 70:30 (outer)	111.83 ± 6.23	95.1 ± 1.9

**Table 3:** Mechanical properties of cylindrical and asymmetrical scaffolds determined after incubation of the scaffolds in artificial saliva (in vitro degradation test) and with p-MSCs (scaffold cellularization).

	Young’s Modulus (MPa)	Yield Stress (MPa)	Ultimate Tensile Strength (MPa)	Fracture Point (MPa)	Elongation at Break %
In vitro degradation test in artificial saliva (37 °C)					
Pig’s Esophagus	0.27 ± 0.01	2.55 ± 0.13	4.39 ± 0.03	3.17 ± 0.56	200 ± 18
Cylindrical t_0_	0.97 ± 0.10	2.87 ± 0.06	6.09 ± 0.21	3.89 ± 0.2	260 ± 33
Asymmetric t_0_	0.39 ± 0.07	1.91 ± 0.10	6.22 ± 0.01	6.13 ± 0.2	273.1 ± 28
Cylindrical t_7_	0.52 ± 0.07	3.44 ± 0.1	5.18 ± 0.02	3.92 ±0.7	131.8 ± 32
Asymmetric t_7_	0.65 ± 0.02	3.7 ± 0.1	5.07 ± 0.24	5.05 ± 0.06	215 ± 11
Cylindrical t_14_	0.73 ± 0.2	2.35 ± 0.04	3.13 ± 0.08	2.86 ±0.05	119 ± 25
Asymmetric t_14_	0.45 ± 0.08	3.15 ± 0.09	4.73 ± 0.02	4.72 ± 0.05	196 ± 14
Scaffold incubation with p-MSCs (37 °C)					
Asymmetric t_7_-ctrl in DMEM	1.1 ± 0.03	6.15 ± 0.3	10 ± 0.03	7.96 ± 2.99	121 ± 23
Asymmetric t_7_-p-MSCs	0.25 ± 0.09	1.9 ± 0.06	3.56 ± 0.23	2.54 ± 0.09	227 ± 32
Asymmetric t_14_-ctrl in DMEM	0.35 ± 0.03	4.67 ± 0.2	5.11 ± 0.5	2.36 ± 0.7	61 ± 15
Asymmetric t_14_-p-MSCs	0.16 ± 0.03	4.33 ± 0.23	5.53 ± 0.02	5.48 ± 0.12	177 ± 17

## Data Availability

The data presented in this study are available on request from the corresponding author.

## Supplementary Materials

The following supporting information can be downloaded at: https://www.mdpi.com/article/10.3390/pharmaceutics14020252/s1, Figure S1: DAPI staining (A1–B1) of asymmetrical patch cellularized by vertical seeding method after 14 days of incubation. SEM analysis (A2–B2) of asymmetrical patch cellularized by vertical seeding method after 14 days of incubation; Figure S2: (a) polymeric asymmetrical scaffold; (b) 8 mm portion; (c) junction; (d) 10 mm portion; Table S1: Component of Shellis’s artificial saliva.
