# Supplementary material for: Engineered Full Thickness Electrospun Scaffold for Esophageal Tissue Regeneration: From In Vitro to In Vivo Approach
Source: Pharmaceutics. 2022 Jan 21;14(2):252. doi: 10.3390/pharmaceutics14020252 (PMC8876746; doi:10.3390/pharmaceutics14020252)
Supplement: Supplementary file 1 [file pharmaceutics-14-00252-s001.zip › pharmaceutics-1547096-supplementary.pdf]

Supplementary materials: Engineered Full Thickness Electrospun Scaffold for Esophageal Tissue Regeneration: From In Vitro to In Vivo Approach

The artificial saliva was prepared according to the formulation developed by Shellis in 1978 containing not only ions, vitamins, aminoacids but also growth factors, mucin and enzyme.

Table S1: Component of Shellis's artificial saliva

Component of Shellis's Artificial Saliva	mmol/l
$CaCl_2$	1.4
$MgCl_2$	0.2
KH_2PO_4	2.6
K_2HPO_4	2.6
Na_2HPO_4	2.6
NaCl	2.6
KCl	15.6
NH_4Cl	4.4
KSCN	2.3
$NaHCO_3$	6.4
Urea	2.9
Mucin (g/l)	2.5
Albumin (μmol/l)	0.4
α-Amylase (Somogyi units)	3×10^5

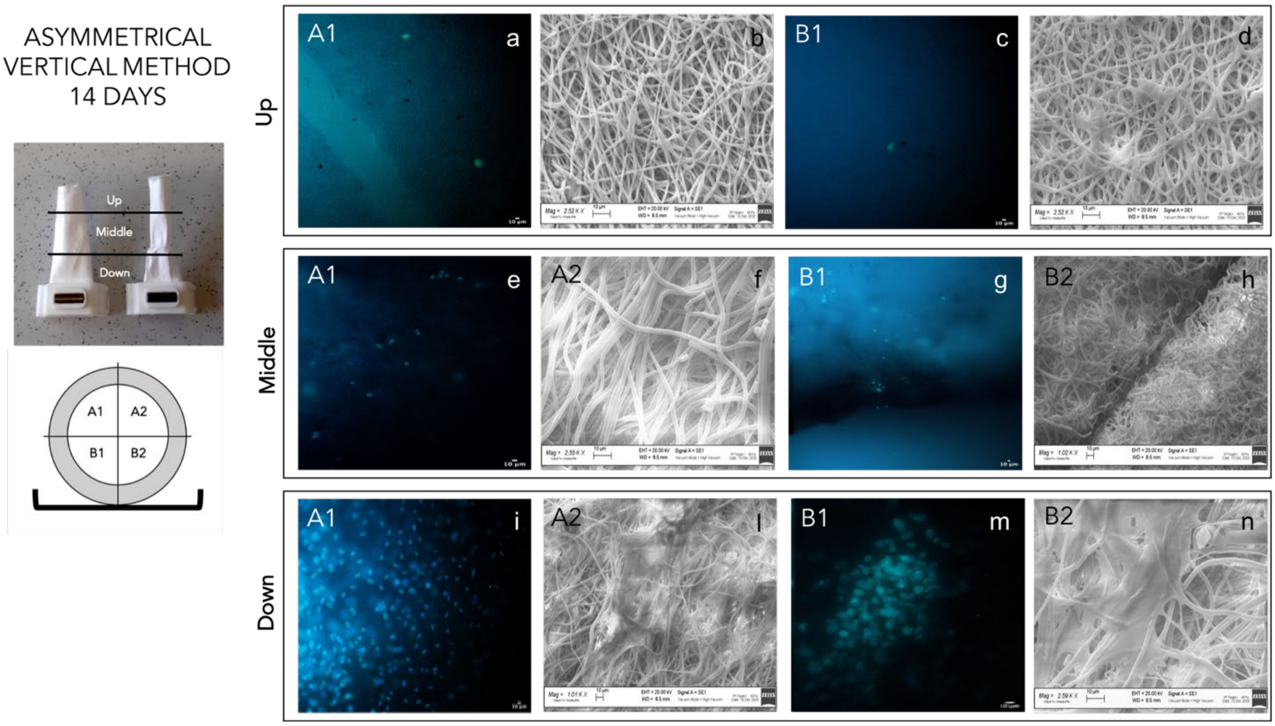

Figure S1: DAPI staining (A1-B1) of asymmetrical patch cellularized by vertical seeding method after 14 days of incubation. SEM analysis (A2-B2) of asymmetrical patch cellularized by vertical seeding method after 14 days of incubation. (**a**) up A1 portion DAPI staining; (**b**) up A2 portion SEM image; (**c**) up B1 portion DAPI staining; (**d**) up B2 portion SEM image; (**e**) middle A1 portion DAPI staining; (**f**) middle A2 portion SEM image; (**g**) middle B1 portion DAPI staining; (**h**) middle B2 portion SEM image; (**i**) down A1 portion DAPI staining; (**l**) down A2 portion SEM image; (**m**) down B1 portion DAPI staining; (**n**) down B2 portion SEM image.

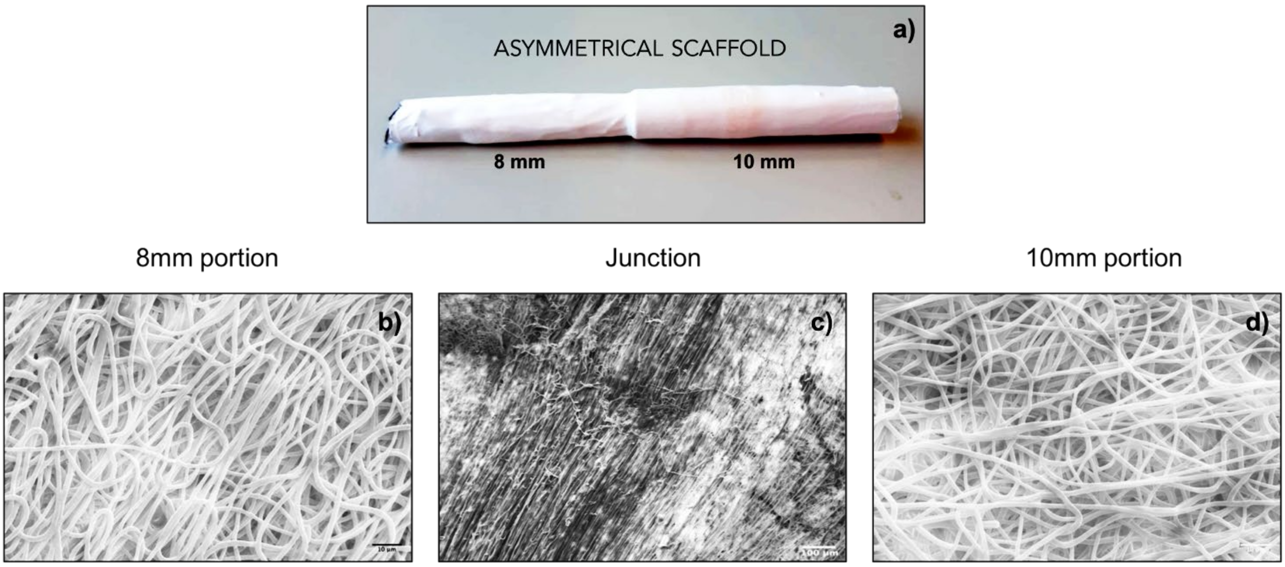

Figure S2: (**a**) polymeric asymmetrical scaffold; (**b**) 8mm portion; (**c**) junction; (**d**) 10mm portion
